# “This is all for *high heid yins*, what about Scottish women?”: Clashing expertise, fraught collaborations, and the 1983 Scottish Women’s Health Fair

**DOI:** 10.1177/09526951261452839

**Published:** 2026-09-01

**Authors:** Karissa R. Patton

**Affiliations:** 1 University of Edinburgh, UK

**Keywords:** expertise, feminisms, grassroots organising, politics of collaboration, women’s health

## Abstract

In May 1983, two women’s health events were held in Scotland. The first was the Women and Health Conference (WAHC), co-organised by the Scottish Health Education Group (SHEG) and the World Health Organization (WHO) European Region. This conference was held in the town of Peebles, Scotland and hosted approximately 200 invited delegates, largely from Europe and North America. The second event, the Scottish Women’s Health Fair (SWHF) was designed in direct response to the “closed and elite” WAHC and welcomed over 2,000 women from across Scotland to Edinburgh’s city centre at the end of May. Using the archival materials from the SWHF and the WAHC and oral history interviews with SWHF organisers, this paper analyses the intersection and divergences between the grassroots approach of the SWHF and the national and global health agendas of the WAHC. I also explore the personal and collective ramifications of these fraught collaborations. While the SWHF ultimately remained “associated” and occurred alongside the conference, the organisers of the fair designed their event in opposition to the WAHC’s top-down, medicalised, and universalised women’s health agenda. In theory, these were two associated events and both had support from the SHEG. But in reality, the history of these two women’s health events underscores deep professional and feminist divides about how to approach women’s health issues and who was best positioned to effect change.

On the weekend of the 27–29 of May 1983, over 2,000 women from across Scotland flooded Edinburgh’s city centre, or “New Town,” to attend the Scottish Women’s Health Fair (hereafter, SWHF or the fair). The fair was planned and run by an organising committee of people involved in the women’s health movement through grassroots organising, public health programming, healthcare provision, health research, policymaking, or, in many cases, a combination of these roles.^1^ Sponsored by the Scottish Health Education Group (SHEG), the organisers of the fair offered a packed programme of talks, workshops, film screenings, and information stalls. The SWHF was not the only women’s health event that occurred in Scotland at the end of May 1983. From the 25–27 of May that year, the World Health Organization (WHO) European Region, with the collaboration of the SHEG, held a Women and Health Conference (hereafter, WAHC or the conference) in the town of Peebles, about 35 km south of Edinburgh in the Scottish Borders. While these events overlapped in timing, sponsorship, and the topic of women’s health, over the course of 1982 and 1983, the collaboration between the SWHF and the WAHC became increasingly fraught. The success of the SWHF in May 1983 occurred despite a turbulent relationship with their sponsors and collaborators. The history of these 1983 women’s health events situates the SWHF as a response to the transnational and professional hierarchies built into the WAHC and, most importantly, as an assertion of local grassroots expertise. In this article, I explore these relationships and conflicts from the perspective of the SWHF organisers, to trouble notions of sisterhood and explore splintering feminist approaches to health and change in the early 1980s.

On the surface, these two events were tied together through a shared goal of bettering women’s health and their collaboration and support from the SHEG; in reality, the SWHF was never part of the WHO’s or SHEG’s plans for May 1983. In 1982, the WHO European Region approached the SHEG about cohosting a conference on women and health in Scotland. Yvonne Bostock, a women’s health organiser and the SHEG employee producing their women’s health information, remembers hearing about the WHO’s visit: “it was like everything we’d worked for was being recognised.”^2^ At her first meeting with the WHO representatives, Yvonne quickly realised that she, and other women’s health activists in Scotland, would have little say in the proposed conference. To appease Yvonne and convince her to work with the WHO, her boss and male colleague at the SHEG offered to sponsor another women’s health event, a health fair, that could be organised by Scottish women’s health activists. As the planning of the two events went on, the different approaches of the respective organising committees contributed to an ever-expanding gap between the SHWF and the WAHC.

Even in the basic programme design and as numbers of attendees at each event demonstrate, the respective organisers had contrasting philosophies on how to effect change. The WAHC limited their audience to approximately 200 invited delegates from across Europe and North America, and featured talks and workshops by researchers, health practitioners, and high-profile activists from the women’s health movement. The WAHC marketed this event as an opportunity to deliberate on the social and health-related issues women faced in society and how institutions could enact change to better women’s health and well-being.^3^ In doing so, the WAHC organisers hoped that the attendees would take information about women’s health inequalities from the conference to effect change from their positions of power within the health, education, and political sectors. Following the conference, the SWHF ran a broad and inclusive programme that reflected the feminist health activism background of the organising committee. The organisers of the fair used community-driven design approaches to centre “ordinary” women’s health experiences and needs, which successfully recruited over 2,000 women from across Scotland to attend the various events and venues of the SWHF. In this sense, the significance of the health fair goes beyond its demonstration of feminist health philosophies in action, as the presence of thousands of Scottish women made the fair a showcase of strength and support for the women’s health movement and its aims in Scotland in the 1980s (Patton, 2025, p. 75).

This article is a history of the SWHF from the perspective of its organisers and, in some cases, specifically of Yvonne Bostock. As historian Jeska Rees has demonstrated, feminists active in the 1970s and 1980s were shrewdly aware of the political act of preserving their history in both archival materials and oral history interviews. Consciousness raising in the late 20th century instilled the act of sharing personal experiences as a powerful political tool. But the reality of misogyny in women’s workplaces, schools, and homes meant that sharing these stories without intention and anonymity could have severe consequences (Rees, 2010, pp. 185–186). The organisers of the SWHF follow this trend in their purposeful sharing of stories, language use, and choice of which stories to share. This politicising of the historical record occurred as the organisers of the fair recorded every absence of SHEG officials at their meetings, every poorly communicated SHEG protocol, every unanswered question about funds in real time.^4^ And in their final reports, they outlined how these small grievances had a large impact on their ability to organise the fair, and yet they succeeded.^5^ Even the act of preserving these records in an official archive demonstrates SWHF and women’s health activist Penny Richardson’s dedication to ensuring the work and impact of the fair would take up space in the historical record. The broader framing of the SWHF as a response to the limited and exclusive WAHC in archival and oral history sources similarly and importantly distinguishes the two health events and their approach. And Yvonne’s story specifically reveals the internal barriers against the fair from men and other women, including fellow feminists, involved in the WAHC. The intentional recording and framing of their lived experiences underscore an important message: the SWHF was successful in spite of their relationship with the SHEG and the WHO, not because of it. This narrative positions the SWHF organisers as major players, equal to the SHEG and the WHO, in the women’s health conversations that occurred in Scotland in May 1983. This careful political archiving and sharing of their experiences of the early 1980s are valuable in broadening historical perspectives beyond the well-known (and, in some cases, well-curated) institutional voices of national and international institutions like the SHEG and the WHO. Relatedly, these sources provide a platform to analyse grassroots and individual actions that have been previously dismissed or eclipsed by these large institutions.

While this is a history of the SWHF, it is hard to remove the SHEG and the WAHC from the story. I did not set out to write a history that so centrally featured the WHO, a global organisation that gets a lot of historical and contemporary attention (Bhattacharya et al., 2005; Cueto et al., 2019; Kanazawa, 2020; Kessler, 1994; Medcalf & Nunes, 2018; WHO, 2008; WHO Regional Office for Europe, 2010). In fact, as Kelly O’Donnell has argued in her work on the Jane abortion clinic, the importance of the SWHF is that “its stars are women” rather than lawyers, judges, or politicians (O’Donnell, 2017, p. 80). But the stories of the WHO, SHEG, and WAHC remained entwined with the story of the SWHF within the archival materials. I consulted materials preserved about the SWHF from the Lothian Health Service Archives, including meeting minutes, promotional materials, administrative records, and the SWHF programme. Documents about the WAHC from the WHO’s archives are also analysed in this research in an effort to consider diverse perspectives about the two events in 1983. However, unlike the variety of files preserved about the SWHF, the files archived about the WAHC by the WHO, including the official conference programme, attendee lists, and report, represent a surface-level institutional record of the conference, with little personal narratives about individuals’ experiences of organising the WAHC. The implication of such a curated and official archival record, in this instance, is that the sources used in this article rely largely on the SWHF organisers’ perspective about the SHEG’s and WHO’s efforts in 1982–1983. For example, the narrative that the SWHF was organised in response to—and even in the face of—the exclusive WAHC was evident in much of the SWHF archival materials and the news coverage about the fair between 1982 and 1983.^6^ More granular notes of discontent between the organisers of the fair and those of the WAHC are evident throughout meeting minutes, reports, and other administrative documents in the SWHF archival files. In these archival files, the organisers of the fair carefully catalogued moments of dissatisfaction with the SHEG and the WAHC organisers, including notes about informational gatekeeping, lack of transparency when it came to budgets and sponsorship, and lapsed communication. These files suggested that there was more than a difference in approach to organising and effecting change between the two events, as the professional relationship between the organisers of the respective events increasingly soured over the course of 1982 and 1983.

Noting this narrative in the archival materials that underscored conflict between the SWHF and the WAHC organisers, I asked oral history narrators to elaborate on the relationship between the SWHF organising committee, the SHEG, and the WHO. (I also reached out to some people who organised the WHO’s WAHC to be interviewed about this history but had no responses.) In general, oral history narrators outlined the fair as a response to the exclusivity of the conference. As part of a broader history of health social movements that responded to patriarchal biomedical, political, and NGO systems (Della Porta & Christou, 2025, p. 3), many of the SWHF interviewees also told me they objected to the fact that the WHO would be holding this conference in Scotland but would not include many Scottish women as delegates.^7^ One particular interview, with former SHEG employee and women’s health activist Yvonne Bostock, was integral to analysing the themes of authority, collaboration, and conflict within and across the two women’s health events. As a SHEG employee and the person who piloted the SWHF, Yvonne was an insider to both the SWHF and WAHC organising. Her professional insight offered rich details about the tensions between the on-the-ground women’s health work in Scotland, patriarchal officials in Scottish health politics, and the WHO’s international health agenda. And her personal reflections underlined how her position at the SHEG placed her in the middle of these broader tensions, which ultimately led to “the beginning of the end of [her] career in health promotion.”^8^

As a feminist oral historian, my aim is to embrace the complexities within the various meanings and collective narratives of the SWHF to recognise and provide nuance on its historical significance. Oral history as a method for sharing and validating women’s experiences has been a hallmark of feminist historians’ methodology since the 1970s (Abrams, 2010, p. 71; Gluck, 2011; Gluck & Patai, 1991; Iacovetta et al., 2018). As Jeska Rees advocates,

feminist historians have a duty not only to record and evaluate the Women’s Liberation Movement critically, but to do so in a way that recognizes the centrality of the personal, the private and the precious to the ideas, achievements and failures of women’s liberation. (2010, p. 182)

Building on my broader work that places grassroots feminist health work as integral to women’s health care in the late 20th century, this analysis of the SWHF and the WHAC demonstrates broader debates about expertise and feminist strategies to improve women’s health in the 1980s. The women who shared their stories in oral history interviews not only shared their memories about the fair itself but of the impact it had in the city and on their own lives—for better and for worse. Following feminist oral history methods and theory, this article does not seek to mine these interviews for historical facts. Rather, the generously shared memories are included here to help understand the significance of the fair on local, national, and global health stages of the early 1980s.

The history of the SWHF and its relationship to the WAHC, understood within these feminist archives and oral histories, underscores professional and feminist divides about how to approach women’s health issues and who was best positioned to effect change. In this article, I explore the historiography of women’s health politics and activism, including work on NGOisation, before exploring the politics within the oral and archival memory about the SWHF and WAHC. From the perspective of the SWHF organisers, gathered from archival and oral history evidence, in this instance, the WHO and SHEG can be understood as international and national interlopers exerting institutional power rather than allies with a common goal. Moving beyond the narrative of sisterhood, the story of the SWHF provides an example of how the divergent feminist approaches and philosophies operating in the 1970s and 1980s had on-the-ground consequences for individual figures and the relationship between global health and grassroots health movements.

## Women’s health politics and movements in the late 20th century

This article places the fraught collaborations between the SWHF and the WAHC in local, Scottish, British, and international contexts of the turbulent health policy and politics of the second half of the 20th century. Following the Second World War, national healthcare systems were introduced in the United Kingdom (UK), and the United Nations (UN) developed their own global health branch, the WHO (Berridge et al., 2011, pp. 181–183; Cueto et al., 2019, p. 2; Millward, 2022, p. 17). During the Cold War era and by the end of the 20th century, critiques of biomedical knowledge systems, approaches, and power (within institutions like the National Health Service [NHS], the WHO, and beyond) grew as intellectuals, like Michel Foucault and Thomas McKeown, and health activists, including decolonial movements, the women’s health movement, mental patient groups, and Black liberationists, critiqued the hierarchical power dynamics in the doctor–patient relationship and within biomedical institutions (Berridge, 1999, pp. 56–57; Cueto et al., 2019, p. 154). At the same time, widespread political and economic shifts in Britian’s international connections and influence caused national moral panic about the strength of the British nation and empire (Thompson, 2011, pp. 7–17, 29, 282). On a global health scale, the rise of decolonised and independent countries that had broken away from their colonial rule shifted the make-up and power dynamics in global organisations, especially the UN and the WHO, from a handful of national superpowers to an increasing number of influential and independent countries (Cueto et al., 2019, p. 154). On a local and national scale, grassroots community health groups, who were unsatisfied with the existing health systems in their communities, created their own health organisations to provide health services and information (Berridge, 1999, pp. 72–73). The community-driven nature of these grassroots health groups, including the women’s health movement in places like the UK, Canada, and the United States (US), sometimes clashed with the established biomedical and political systems of health institutions like the NHS and the WHO (Elizabeth, 2023; Houck, 2023; Kline, 2010; Patton & Wood, 2023; Strimple, 2022). My analysis of the SWHF is one example of the fraught landscape of women’s health politics in the mid to late 20th century that bridges the historiography of global health politics and the splintering of the feminist movement between 1948 and 1990.

By the 1980s, as the broader women’s liberation movement was splintering along lines of race and sexuality, debates among feminist health activists about whether grassroots and institutional approaches to change could work together effectively splintered the movement along lines of professionalisation (Elizabeth, 2023; Houck, 2023; Kline, 2010; Patton & Wood, 2023; Strimple, 2022). From the early 1970s, feminists categorised their divergent philosophies and methods for change. Some feminists (liberal feminists) aimed to change society by “breaking the glass ceiling” and obtaining positions of authority in order to change power dynamics “from within” existing institutional structures (Nicholson, 1997, pp. 3–4). Radical feminists, Marxist feminists, and socialist feminists—in their own distinct ways—charged that the only way to change society and break down oppressive power structures was to dismantle existing institutions and create new systems of politics, care, and knowledge based on equality of gender and class (Nicholson, 1997, pp. 2–4). Beyond these broad categories of feminist philosophies, the feminist movement “necessarily” splintered along identity lines, allowing Black, Indigenous, Chicana, and lesbian feminists to form their own organisations throughout the late 1970s and the 1980s (Houck, 2023, pp. 9, 219; Roth, 2004, pp. 2–3, 40–42). Different philosophies and splintering along identity and professional lines also changed the make-up of the women’s health movement across different nations during this time. As Dudley-Shotwell and others have shown, many feminist self-help groups and health centres “argued over the extent to which self-help activists should attempt to work with mainstream medical institutions or create their own separate institutions” (Dudley-Shotwell, 2020, p. 7; see also Houck, 2023, pp. 83–85; Patton & Wood, 2023, pp. 194–195; Quéré, 2021, pp. 341–342; Ruault, 2021, pp. 321, 323–324; Tafuro, 2024, pp. 90–91). Judith Houck’s work similarly demonstrates that other feminist health groups tried to work across grassroots and health professional spaces but found it difficult to “fit into a system that required state licensure, federal funding, and medical cooperation” (Houck, 2023, p. 85).

Tensions surrounding feminist approaches to change and the role of professional women and organisations in the women’s health movement played out on local and global stages throughout the end of the 20th century. The UN and WHO were often part of these debates. Throughout the 1970s, some feminist scholars and health groups embraced the WHO’s broad definition of health to bolster their claims that social change was key to improved health (Dixon-Mueller, 1993, p. 139; Patton, 2021, p. 81). However, many feminists in places like Canada, the UK, and the US also felt the WHO was not advocating for reproductive rights strongly enough (Cueto et al., 2019, p. 159). Maud Anne Bracke’s work describes how some grassroots groups underwent “a process of gradual professionalisation and ‘NGO-isation’” to be recognised as experts within the UN (Bracke, 2023, p. 825). While the UN’s 1994 Conference on Population and Development (Cairo) and 1995 Women’s Conference (Beijing) have been used to sign-post the emergence of a global reproductive rights movement, Bracke underscores the significant contributions of feminist groups in establishing a global reproductive rights movement before the 1994 and 1995 UN conferences. As Bracke has shown, the grassroots and, sometimes, “militant” feminist groups who laid this foundation had to navigate expectations of professionalism to be taken seriously within respective NGOs (2023, pp. 823–825). This necessity of “NGO-isation,” as Brake explains, can be applied beyond just the UN, as her work demonstrates the uneven exchange of expertise between grassroots groups and NGOs.

In line with Brake’s historical analysis, women’s, cultural, and activist studies scholars have shown how an uneven balance of knowledge exchange often occurs between NGOs and activist groups, in the past and present (Choudry & Kapoor, 2013; Fernández-Aballí, 2015; Jad, 2007). Social and grassroots movements are often encouraged to connect with NGOs that have funding and resources to implement wide-spread programmes to support identified common causes. The NGOs benefit from these relationships as they incorporate certain activist language and concepts to bolster support for their programmes. While NGOs do sometimes collaborate with grassroots groups, many of these activists ultimately negotiate their approaches to fit within the NGO’s standards of professionalisation. And a lack of reciprocity remains in these relationships, as activists in general cannot openly commission NGOs to take up their causes. In this sense, the rhetorical power of the global institution often eclipses the more radical, complex, or innovative endeavours of local grassroots groups.

Feminist political studies scholars offer specific analyses of the complex relationship between grassroots women’s groups and global NGOs, including the UN and the WHO. Annelise Riles (2002) and Nicole George (2012), for instance, have shown how activists in the women’s movement have faced power struggles for information sharing and policy development in collaborations with professionals in national and global organisations (George, 2012; Riles, 2002, p. 290). George in particular outlines the “costs that women’s movements incur as they become ‘professionalised’ through their engagement with formal political institutions” (2012, p. 2), as she traces how Fijian women’s groups negotiated their mandates with global NGO and national policymakers following decolonisation. In the same vein, some scholars have directly critiqued the WHO for “avoiding feminist practice in health emergency response” and increasing the divide between the grassroots gender activism and global policy work (Davies & Wenham, 2023, p. 216). Deborah Mindry’s concept of the “imagined feminised ‘global family’” offers a framework to tease apart the disconnect between local and global women’s organisations (2001, p. 1189). In her analysis of interactions between women’s work in NGOs and grassroots groups in India, Mindry argues that women from charitable NGOs, largely based in the Global North, framed their work as moralised, “feminized and naturalized obligations” to help their sisters in the “global family” (Mindry, 2001, p. 1193). These same women, as Mindry explains, maintained that projects, and by extension the individuals, they supported had to be deemed “deserving of help” (Mindry, 2001, p. 1193). By extension, in the face of critique from local women, the women in the NGO providing the aid “were also critical of poor, uneducated women who, in their view, often did not cooperate in their own empowerment” (Mindry, 2001, p. 1195). Taken together, this body of work reveals that even when working with grassroots groups, global policy-makers frame NGOs as the best entities to set agendas and provide the most effective route to social change. This framework establishes these collaborations within a hierarchy of expertise from the start, with the expectation that grassroots groups will adjust their goals and approaches to align with the priorities of NGOs.

Within the context of early 1980s Scotland, both public health and feminist community organisations grew more concerned about national health outcomes than global health initiatives. The “sick man of Europe” or the “British disease” had been used throughout the 1960s and 1970s to describe the poor economic and health outcomes of Britain as well as its wavering place on the global stage (Allen, 1979; Crafts, 2012, 2018; Nannestad, 1991). By the 1980s, Scotland in particular took on the nickname of “sick man of Europe” in reference to “the highest mortality in Western Europe among working age men and women,” as well as high rates of heart disease, liver disease, and lung cancer, for example (Haddow et al., 2014, pp. 3, 5–6; McCartney et al., 2011, p. 756). Yvonne Bostock and others working in public health services in Edinburgh remember the SHEG’s large-scale antismoking and heart disease awareness campaigns during the late 1970s and early 1980s. Public health researchers and women’s health activists Yvonne and Amanda Amos also remembered that all of these major campaigns targeted men, despite the fact that women in Scotland also had the poorest health outcomes in the UK.^9^

To address these particular health, political, and cultural contexts, feminist groups in Scotland similarly argued for nationally specific reproductive rights campaigns that addressed Scottish women’s local realities (Browne, 2014, p. 64; Hay, 2021, pp. 596–597; Patton, 2025, p. 75). Feminist health activists in Scotland often encountered different legal and policy challenges, such as abortion access, and employed specific strategies, such as working closely with trade unions, than their counterparts in England and Wales (Hay, 2021, p. 601; 2025, p. 653; Sheldon et al., 2022, pp. 39–40, 48). Scottish feminist organisations often had to combat a conservative “medico-moral” framework that championed “traditional” heterosexual marriage and family formations in Scotland (Davidson & Davis, 2012), pp. 294–295; see also Brown, 2012; Hay, 2025; Lynch, 2021; O’Neill, 2018; Patton, 2025). Kristin Hay’s work on the Scottish Abortion Campaign (SAC) offers one example of how Scottish activists prioritised Scotland-specific issues over some UK-wide campaigns. As Hay explains, the SAC emerged as an independent branch from the broader UK National Abortion Campaign: “responding directly to the needs of Scottish women and their access to legal abortion, they had their own Steering Committee, affiliations and campaign strategies” (Hay, 2021, p. 596). Building on the work of Sarah Browne (2014), Hay’s work on the SAC illustrates “a distinctively Scottish feminist identity” forming in the late 1970s and early 1980s (Hay, 2021, p. 609).

In 1982 and 1983, the nationally driven approach of feminists and public health groups collided with the global mandate of the WHO. It was the SHEG’s national public health campaigns, including Yvonne’s women’s health projects, that drew the WHO’s attention in the early 1980s.^10^ But, perhaps ironically, the nationally specific concerns of women’s health activists did not transfer into the agenda for the WAHC. Beyond the local–global divide, the decision not to include Scottish women’s health groups and activists in the WAHC suggests that the SHEG and the WHO did not see Scottish women as equal collaborators. Alongside the work of Bracke (2023), Mindry (2001), and others (Choudry & Kapoor, 2013; Fernández-Aballí, 2015; George, 2012; Jad, 2007; Nott, 2002; Riles, 2002; Sawer et al., 2023; Wenham & Davies, 2022), this article flips this script and positions the grassroots organisers of the SWHF, rather than the WHO or the SHEG, as the key change-makers in this history. By centring the perspectives of the SWHF organisers and taking seriously their archived complaints about the SHEG and the WHO, I challenge the narrative that the national and international institutions were better situated to effect change in society. The success of the SWHF alone demonstrates the impact of the fair for women across Scotland, as well as a showcase of strength in the Scottish women’s health movement (Patton, 2025, pp. 74–75). But the stories recorded in this article about the trials of collaboration demonstrate that it was not just misogynistic political or medical players who hindered grassroots feminist organising. Rather, I explore how institutionalised approaches, professional boundaries of expertise, and divergent philosophies about effecting change fuelled division across two seemingly aligned women’s health events.

## “You should really get this story from Yvonne”: The personal, the political, and the oral histories of the SWHF

This research relies on the administrative files of the SWHF donated by one of the organisers and five oral history interviews with some of the organisers themselves. Initially, during the spring of 2024, I was able to find and interview the following SWHF organisers: Amanda Amos, Jane Jones, Adrienne Sillar, Gica Loening, and Yvonne Bostock. Amanda Amos was a genetics student and public health postgraduate who was an active women’s health activist and became a leading researcher on tobacco control, women, and youth from the 1980s onwards. Women’s liberation and natural childbirth activist of the 1970s, Jane Jones ran women’s health programmes from a community health project in Pilton, Edinburgh from the 1980s into the 21st century. Adrienne Sillar was a physiotherapist and women’s health activist in the 1970s and 1980s, an organiser of the SWHF, and, later, ran the Well Women Centre in Gorgie, Edinburgh. Gica Loening was part of the women’s peace and women’s health movements in the 1980s, and carried on the legacy of the SWHF through her work at the Well Women Centre in Gorgie in the 1980s and 1990s. These four oral history narrators shared memories about the planning committee, the events of the fair itself, and the impact the SWFH had on women’s health in Edinburgh and beyond. Finally, I spoke with Yvonne Bostock, who was a women’s health activist and research officer at the SHEG in the late 1970s and early 1980s. Yvonne was central to the operation of both the SWHF and the WAHC through her role as the women’s health expert at the SHEG in the early 1980s. Her memories of this time provided a more complex story about the origins of the SWHF and deeper reflection on the tensions between the grassroots organisers and the authority figures at the SHEG and the WHO. Her interview is central to understanding, from her own and the collective SWHF perspectives, the fraught collaboration between the two women’s health events in Scotland in May 1983.

Many of the women I interviewed clearly remembered that the SWHF was designed in contrast to what they saw as the shortcomings of the WAHC. Jane Jones, for example, remembered,

the WHO were going to have this Women’s International Health [conference] … maybe the health fair came first? That’s right, we were told there was going to be a health [conference]. And then, because there were all these seeds of women[‘s groups] [who said] “well, we want a community one.” So we started planning our health fair.^11^

Others, like Adrienne Sillar and Amanda Amos, remembered that the collective upset about the WAHC sparked the idea of the SWHF. Adrienne explained,

when the idea for the women’s health fair came up, which, as you know, was because the World Health Organization were having a big international conference in Peebles. And people thought in Edinburgh, “this isn’t going to be relevant, really, to the local women.”^12^

Similarly, Amanda told me,

the WHO was [organising] the first ever women’s health [conference], and Ilona Kickbusch was heading that up. And it was going to be in Peebles. And Yvonne was the Scottish link through SHEG. And it then … this is all before I got involved, that, I suppose, women involved in the women’s movement started saying, “well, hang on, this is all for *high heid yins*, what about Scottish women? How is our health going to benefit from this?” And after that, emerged the idea of [the] Scottish Women’s Health Fair.^13^

Amanda’s recollection clearly notes frustration from the local women’s groups about the WHO and SHEG’s approach to organising the conference. Her use of “*high heid yins*” in her description of events is of particular note, as the Scots’ phrase is used literally to describe people in positions of authority, but is also often used sarcastically to refer to someone in a position of power who alludes self-importance or who is out of touch with others. Her invoking of this Scots’ term casts the story in a particular light for Scottish insiders: The *high heid yins* of the WHO and SHEG were not just wielding their organisational authority in organising the WAHC, their invitation-only platform demonstrated how out of touch they were with the on-the-ground women’s health work actually occurring in Scotland. Amanda’s recollection of local women’s opinion of the WAHC underlines the tensions surrounding professionalism, expertise, and methods for effecting change between the locally driven grassroots groups and the national and international institutions—SHEG and the WHO.

These memories from Amanda and others aligned with archival materials and news coverage at the time that explicitly framed the SWHF as a response from the Scottish Women’s Health Movement to an external and elite event. Scottish feminist journalists wrote newspaper articles covering the WAHC that presented a local distaste for the WHO and SHEG’s approach to the event (Patton, 2025, pp. 89–90). For example, one article by Shiela McNamara critiqued the WAHC for the fact that only one of the invited delegates was Scottish, and argued that “it was felt that the people most concerned in the matter—women themselves—really ought to have some involvement somewhere.”^14^ This narrative of the SWHF as designed in response to the shortcomings and exclusivity of the WAHC, therefore, was active in framing the need for the fair in 1982 and 1983. And these critiques of the WAHC have been maintained in the recollections of the oral history interviewees.

Despite this strong narrative in the archival materials and the oral history interviews that the SWHF was responding to what many felt was an exclusive and outsider event, most of the interviewees only briefly remarked on the tensions between the two respective events. When asked to elaborate on the relationship between the organisers of the SWHF and those working at the SHEG and the WHO, the narrators often offered vague recollections before directing me to Yvonne Bostock. In Adrienne’s case, she remembered she got involved slightly later than some others and only recalled that Yvonne was central to arranging the sponsorship of the fair. Jane similarly highlighted that Yvonne was the main connection between the SHEG and the SWHF and that she secured the fair’s organising committee’s funding and printing privileges at the SHEG. She also reflected that Yvonne was likely “alone” at the SHEG in her support of the fair: “She probably had a hard time in the organisation of it, actually because I don’t know if SHEG were all that supportive.”^15^ Amanda explained, “Yvonne, this women’s health conference coming and the women’s health fair, she really put herself out, and in the end, it had consequences for her career.” All of their recollections about the tensions featured broad narratives about the exclusive nature of the WAHC and specific references to Yvonne. Eventually, Amanda told me, “You should really get this story from Yvonne.”^16^

While some interviewees recalled more details than others about the relationship between the SWHF and WAHC organisers, their similar responses in directing me to Yvonne imply a sense of responsibility to allow Yvonne to tell this story. Adrienne, Jane, and Amanda were not censoring information but demonstrating care and solidarity for Yvonne’s lived experience by leaving it up to her to decide what and how much of this story to share. Jeska Rees’s work on oral histories of women’s liberation movements offers a framework to understand these spontaneous but similar responses directing me to Yvonne as part of a feminist politics from the 1970s and 1980s of protecting people’s stories. Underscoring the “highly emotive accounts of women’s experience” in written and oral sources of women’s liberation movements, Rees argues that feminists who were active in the 1970s and 1980s developed careful and thorough ways to share their memories: “Their stories, and the way that those stories are told, are of great importance to them” (Rees, 2010, p. 180). Most of the women I interviewed invited me into their homes to conduct the interview and shared not only their memories but their personal archives of the SWHF and other women’s health work in which they participated during the 1970s–1980s. This level of trust was likely built over time as each individual explicitly or implicitly “vouched for me” by passing on my contact information to their friends and colleagues—a common benefit of snowball interview recruitment methods. Despite this initial trust in me and my work on the SWHF, when it came to specific discussions about the tensions between the SWHF and the WAHC organisers, each interviewee referred me to Yvonne Bostock. The oral history narrators evoked a feminist practice of what Rees describes as a political and personal “guarding” of the more personal and emotional aspects of Yvonne’s story (2010, p. 186).

When I spoke with Yvonne, she began by probing me about how much I knew about the tensions between the SWHF and the WAHC. She asked whom I had spoken with, what others had shared with me in their interviews, and what I had found in the archives. And after I had provided her with the context of why I wanted to explore more about the health fair and that most of the women I had already interviewed directed me to her, Yvonne began to share her story, starting with “the background to it all.”^17^ Reflecting on this interaction, with Rees’s work in mind, this was likely a test of trust and perhaps an investigation of which “side” of the story I had already heard (2010, p. 184). Once I interviewed Yvonne, I understood why the other interviewees had placed such importance on her perspective. Her unique position of working at the SHEG, working with the WHO on the WAHC, and working with the SWHF organisers meant she witnessed the events in 1982 and 1983 and has an insider perspective across the three organisations. Using her interview and, in some places, media accounts of the events, the paragraphs below share Yvonne’s story at length as a personification of the professional tensions and personal ramifications of the fraught collaboration between the SHEG, the WHO, and the SWHF in the early 1980s.

Yvonne started by telling me how much she loved her job at the SHEG and how she had valued working somewhere where she could apply the knowledge and skills she had gained from her women’s health consciousness raising work and research from the 1970s and 1980s. When she joined the SHEG, she began expanding the Scottish public education pamphlets beyond issues about men’s health or perceived male-specific health conditions such as heart disease.^18^ She created pamphlets, booklets, and health promotion programmes for radio about women’s health topics like menopause, women and smoking, and contraception.^19^ In the early 1980s, her hard work and connections with the women’s health movement culminated in the SHEG-produced health book for Scottish women *Well Woman: A Guide to Women*’*s Health* (1983).

In practice, Yvonne brought her activist and professional lives together in her work at the SHEG (Patton, 2025, pp. 79–80), but the separation of the WAHC and the SWHF pressured her to split her time, labour, and allegiances. By 1982, the SHEG’s health promotion work, and Yvonne’s women’s health publications and media successes, had caught the eye of the WHO European Region. When Yvonne heard about the WHO wanting to work with the SHEG on a conference for women and health, she “thought, ‘wow, how exiting.’”^20^ Yvonne recalled her first meeting with the WHO representatives, when it became clear that the collaborative WHO–SHEG WAHC would be for invited international delegates only. Yvonne told her boss and WHO representative Ilona Kickbusch,^21^ “we’ve got to do a Scottish conference first and next year, we’ll do the international one.”^22^ In her oral history interview, she told me she argued for a Scottish conference that would acknowledge the trail-blazer women in Scottish women’s health promotion. She told her boss and the WHO representatives that they could not leave these women out of an event on women’s health in their own backyard. But her suggestion was immediately shut down. She said,

I stormed out … I said, “I can’t do it. No, it’s just not right, I’m sorry, what is waiting a year? Why do we have to play and jump to WHO’s tunes? For goodness’ sake, think of us” … I still don’t get it.^23^

Yvonne’s boss eventually offered a compromise: Yvonne would help plan the WHO–SHEG WAHC, and the SHEG would also fund a women’s health fair that local women would plan and run in Edinburgh. Yvonne said she felt defeated and dismissed. She formed a SWHF organising committee of local women’s health experts and became part of the planning committee for the WHO–SHEG conference. She said,

I swallowed my pride and got involved in all the administrative organisation of the whole thing [WAHC], not with a passion, quite the contrary really because I felt I’d let these women down that I couldn’t make that [a Scottish conference] happen.^24^

Over the course of 1982 and the beginning of 1983, Yvonne worked with the WHO staff to plan the WAHC and advocated on behalf of the SWHF committee from her position at the SHEG.

At the same time, Yvonne’s work on the *Well Woman* book became a national controversy. She combined all the research she had done on various women’s health topics during her time at the SHEG and wrote a women’s health handbook, which included information for women about different NHS services, women’s bodies—including a section called “Your Intimate Body Parts”—alongside periods, menopause, contraception, and mental health (Patton, 2025, pp. 80–81). The publication date of *Well Woman* was planned to accompany a BBC series under the same title that expanded on the media public health materials Yvonne had created during her time at the SHEG (Vaughan, 1983; Veitch, 1983a, 1983b). The content of the book, specifically the parts that discussed women’s bodies and sexuality and featured a drawn image of a vulva, vagina, and clitoris, upset several chief medical officers across Scotland.^25^ In early 1983, approximately 36 hours ahead of its distribution and with £14,000 already spent on its production, Chief Medical Officer John Reid from the Scottish Home and Health Department demanded the *Well Woman* book be rewritten. Government officials claimed the content was “too strident,” “overconcerned with sexuality,” “smacked at women’s lib,” and was “anti-doctor” (Veitch, 1983a, p. 2; see also Vaughan, 1983). According to newspaper coverage of the *Well Woman* censorship, Director of the SHEG, and Yvonne’s boss at the time, Stanley Mitchell agreed with the government’s critiques of *Well Woman* despite his initial approval for the book to go to print. As one reporter wrote, Stanley Mitchell “confirmed that officials had said it was over-concerned with sexuality. ‘It is a criticism I made of it myself’ Mr Mitchell added” (Veitch, 1983a, p. 2).

With the SHEG director and the Scottish chief medical officer’s disapproval, Yvonne faced backlash about the *Well Woman* book both internally at the SHEG and more broadly. In the newspaper coverage of the censorship of *Well Woman*, Stanley Mitchell claimed that it was his role as director of a “government agency” to have the Home and Health Department sign off on the *Well Woman* book (Veitch, 1983a). The SHEG was a civil-servant-run department linked to both the National Health Service Scotland and the Scottish Home and Health Department, but there was little precedent for seeking official government department approval of publications at that time. Yvonne’s previous work on women’s health promotional materials and programmes had not been subject to approval by the Home and Health Department before. And Stanley Mitchell’s predecessor as director of the SHEG, David Player, often upheld the SHEG’s intellectual autonomy as a research-based public health department.^26^ In fact, David Player had signed off on the publication of the *Well Woman* book before he left the SHEG to become director of the Health Education Council (England and Wales equivalent of the SHEG) (Veitch, 1983b). As Stanley Mitchell admitted he agreed with the Chief Medical Officer’s critiques of the *Well Woman* book, it is possible that he sought approval from the Scottish Home and Health Department knowing the high possibility of stopping publication (Patton, 2025, p. 81).

The controversy surrounding the *Well Woman* book and Yvonne’s fraught relationship with the WHO European Region came to a head at the WAHC. While planning the conference, the WHO representatives working with Yvonne learned about the censorship of the *Well Woman* book. Despite Yvonne’s protest to bring up the issue of the *Well Woman* book at the conference, the issue was raised in a “specially convened workshop on censorship” at the WAHC (Nelson, 1983). The organisers of the special workshop stated in a press release:

This workshop is strongly opposed to the arbitrary censorship of health education material written by lay and professional women for other women. It appears that this is precisely what happened in the case of the booklet to accompany the BBC series *Well Woman* which was to be produced by SHEG. … we deplore the fact that two of the men involved in the suppression of this booklet spoke at the opening session in support of the aims of the conference yet did not stay to hear what women had to say. (Nelson, 1983, p.1)

According to the WHO’s archival records from the conference, Chief Medical Officer John Reid and SHEG Director Stanley Mitchell both spoke at the opening ceremonies of the WAHC.^27^ Yvonne remembers,

at the end of one day [of WAHC], the team of organisers got together and said, “I think we need to do something about the *Well Woman* book” and I said, “what do you mean ‘do something’, what are you going to do?” “I think we should raise it at this conference.” “Raise it?” “I think we should point out what’s happening in terms of how it’s been blocked.” And I went, “no, don’t do that here, this is something I have to deal with diplomatically.”^28^

This special workshop was not listed in the WAHC provisional programme or in the other planning documents available in the WAHC archival materials, so it is likely that this workshop was added to the programme at the last minute and in response to the presence of John Reid and Stanley Mitchell at the opening ceremonies after their direct involvement in the *Well Woman* book criticism and censorship. Those who led this workshop and spoke out against the censorship of the *Well Woman* book at the WAHC also likely positioned themselves in support of Yvonne’s hard work at the SHEG and on the *Well Woman* book in particular. But the presence of several civil servants from the Scottish Home and Health Department and the regional (Lothian) Health Board at the conference, including John Reid and Stanley Mitchell,^29^ put Yvonne in a situation where she felt she could either defend herself and the credibility of the *Well Woman* book or keep her job at the SHEG.^30^

Despite her objections, Yvonne remembered the *Well Woman* book being brought into the conversation at the conference when someone began “criticising the Scottish government and saying this [the censorship of *Well Woman*] was inappropriate.” Yvonne continued to explain that this critique was “followed by somebody, a civil servant, standing up and defending their position. I can’t remember exactly what he said but it was a reflection on me personally.”^31^ Later she recalled,

this guy is standing up and saying, “well, it really has to be rewritten, it’s inappropriate.” And there was a voice in my head saying, “sit down, sit down. Yvonne, sit down, this is going to cost you your job.”… And I stood up and I said, “our job is to give women information on which to base their health decisions, live healthy lives, that is all we’re trying to do here and yet it’s been blocked and blocked by male doctors.”^32^

This exchange was covered the next day on the front page of the Scottish national newspaper *The Scotsman*, which recorded, “There were gasps from the audience when Mr Stanley Mitchell, director of the Scottish Health Education Group, and his research officer, Ms Yvonne Bostock, contradicted each other from the platform” (Nelson, 1983, p.1). At the centre of this tense political situation, Yvonne advocated for uncensored information about women’s health in the face of her boss and other major players in the SHEG and the Scottish Home and Health Department. The Monday following the conference, Yvonne showed up to work to find an empty “in-tray” and a message from Mitchell letting her know she “was not to go to any committee meetings, anything where I represented [the SHEG].”^33^ This approach, called constructive dissmissal, often forced an employee to quit rather than be fired to save the institution controversey. When Yvonne recalled her constructive dismissal at the SHEG, she said she had no choice but to resign from a job she “loved” and “cared about.”^34^

While many interviewees had explained that the SWHF was a response to the WAHC, Yvonne’s story, from her own words and the archival materials, reveals a much more complicated narrative about the origins and organisation of the SWHF. Oral history scholars, such as Alessandro Portelli and Lynn Abrams, have argued that how an interviewee tells and retells their story captures more than just historical facts but information about the interviewee’s own sense of self, their values, their relationships (Abrams, 2010, p. 47; Portelli, 2009, pp. 50–52). In the case of the SWHF, the initial suggestion of the fair seems to be of little importance for Jane, Adrienne, and Amanda, whose efforts were focused on actually providing a platform for Scottish women’s voices and concerns in the face of the exclusive WAHC. As such, their experience organising the fair at the time, as reflected in the strong memories they have about it now, prioritised the success of the fair and the inclusion of Scottish women in the design and invitation as an alternative to the WAHC. For these narrators, they saw the SWHF as a huge success that influenced their careers and activisms going forward. Conversely, the details of when and why the fair was first suggested are significant to Yvonne’s lived experience and how she reflected on the events of 1982–1983. She recalls these events as ending her health promotion career and reflects on the origins of the fair as an attempt by her boss to placate her enough that she would work with the WHO staff to organise the WAHC.

The fact that the initial suggestion to hold the health fair was not from grassroots groups but from the SHEG does not negate the immense community organising and labour to create a Scottish women’s health platform in May 1983. And the organisers of the health fair did respond to what they saw as the exclusive nature of the WAHC by ensuring that they designed the fair from the bottom up, with Scottish women’s input and providing opportunity for a wide base of participation (Patton, 2025, pp. 85–88). Beyond the inspiration for the fair, this story offers a zoomed-in view of the tensions surrounding lay and professional expertise, grassroots and global health initiatives, and various feminist approaches to effecting change in the early 1980s.

## From within or outwith?: The SWHF, the WAHC, and feminist approaches to change

As Yvonne reflected on the WAHC and the SWHF, she pointed out that the circumstances that led to the end of her career in public health were not only linked to men like Stanley Mitchell and John Reid but to other women organisers of the WAHC. She saw the women WHO officials who rejected her ideas about a Scottish conference and the women who rebuffed her protests and organised the special workshop at the conference itself as key players in creating, or at least contributing to, the tensions between the SWHF and the WAHC organisers. She told me, “And what was depressing about it was it wasn’t just men, it was women’s … egos.”^35^ Her recollections align with the SWHF meeting minutes and reports that highlight discontent between the professional organisations and the grassroots health fair beyond Yvonne’s personal experiences. The archived materials and oral histories of the SHWF and the WAHC reveal that certain feminists upheld hierarchies of power and expertise when it came to women’s health.

The SWHF organisers articulated deeper, divergent approaches to women’s health between the WAHC and the SWHF beyond their objections to the “closed and elite conference.”^36^ In their final report in 1983, the organisers of the fair stated that from the early planning stages, they noticed “an interesting difference emerged between their [SHEG–WHO’s] way of looking at women and health and ours.”^37^ They critiqued both the WHO’s and the SHEG’s background papers that were written in 1982 about women and health as a justification for the conference. The SWHF organisers specifically took issue with the SHEG and WHO’s focus on the “importance for health educationalists of making use of the caring role of women by directing information and education at them in the hopes of improving the general standard of health.”^38^ The SWHF organisers’ critique that the WAHC papers were speaking at women rather than bringing women into the conversation to solve problems reinforced their opinion that gender and class imbalances were embedded into healthcare systems, including institutions like the WHO and the SHEG.^39^ In response, they wrote their own background paper and published it in a special issue of the *Edinburgh Women*’*s Liberation Newsletter* in November 1982.^40^ In the paper, they stated,

In our view, women can provide the spearhead for this change. The qualities of giving, sharing and compassion which tended to render women vulnerable are in fact strengths which can readily be harnessed to create a positive and healthy society.^41^

They ended their paper by highlighting the SWHF as an opportunity to “mobilise” and “make significant moves forward.”^42^ By writing their own background paper in 1982, the SWHF organisers staked their claim to the local and national women’s health stage the next year.

As the SWHF organisers outlined the conceptual differences between the design of the fair and the conference, they also grappled with how to maintain their autonomy while being sponsored by the SHEG. The terms of the SHEG sponsorship of the fair are not precisely outlined in the archival records or oral history interviews. But there is a record of an agreement that the SHEG would fund a large part of the fair and that the SWHF organisers would have access to SHEG office resources. With this sponsorship relationship in mind, early meeting minutes from the SWHF archives recorded hopeful statements about cooperation between the SWHF and the SHEG. For example, the organisers of the fair wrote about their plans to “liaise closely with SHEG on publicity … However it was noted that publicity for the Fair needs to be quite different from the Conference.”^43^ A series of events recorded in the SWHF meeting minutes, including SHEG staff failing to attend meetings with organisers of the fair, inadequate access to SHEG resources, and opaque policies about equipment use, demonstrated how quickly, in the words of the SWHF organisers, “the [SHEG] sponsorship, funding and beginnings of autonomy faltered into a grey area.”^44^ The SWHF organisers were not the first activist group to navigate complex, or even hostile, funding situations. Lucy Delap’s historical examination of the business praxis of British feminist magazine *Spare Rib* shows how carefully feminists deliberated on the “challenge of reconciling politics and profit” in 1980s Britian (2021, p. 267). And George Severs’ work on the history of HIV activist organisation ACT UP and their fraught funding relationship with the Wellcome Foundation, and the affiliated Burroughs Wellcome & Co pharmaceutical company, details the strategies that grassroots activists employed to navigate sometimes unsavoury corporate sponsorship. Severs argues that the acceptance of funds from Wellcome was not necessarily an act of alignment with the company on the activists’ part but an example of how they “navigated and manipulated capitalist frameworks to their own political ends” (Severs, 2024, p. 36). The SHEG and the WHO originally approached the SWHF as an appeasement for local activists, rather than a legitimate collaboration. But the SWHF activists held the SHEG to their promise of sponsorship and utilised their capital to assert their own expertise in women’s health.

The organisers of the fair highlighted a lack of trust and cooperation between themselves and the SHEG in their regular meeting minutes and in their final report on the SWHF. Much like the ACT UP activists Severs wrote about (2024), the SWHF activists found ways to utilise the SHEG’s funds and resources that they could access to their own benefit. But the use of SHEG assets did not dissuade them from carefully recording the many sour interactions with them throughout 1982 and 1983. For example, minutes from January 1983 documented the back and forth between Director of the SHEG Stanely Mitchell and the SWHF organising committee about securing one community service volunteer (CSV) to help with the administrative tasks for the upcoming fair.^45^ As a government organisation, the SHEG would have had access to the resources from Volunteer Scotland to request the CVS. However, when members of the SWHF went to Mitchell, he referred “her back to the co-ordinating [SWHF] committee to ask for more volunteer help.”^46^ By early May, less than 3 weeks ahead of the fair, the SWHF minutes recorded issues with accessing SHEG equipment, noting “there is obviously a lot of crossed lines about what Angus from SHEG needs to do for us.”^47^ The final report on the SWHF stated, “the overbearing presence of SHEG staff, often hidden agendas and fight for identity prevented essential establishment of trust and consensus within this group.”^48^ According to the SWHF meeting minutes and reports, these tensions with the SHEG in particular resulted in what the organisers described as unwritten hierarchies and power dynamics between themselves and the SHEG that contributed to produce a “half-hearted structure of communication between the Conference and the Fair.”^49^ The report goes on to describe SHEG employees as “secretive,” especially when it came to the funds allocated towards the SWHF activities. As SHEG staff withheld funds, resources, and information, the organisers of the fair increasingly explored alternative support and resources, which widened the existing gap between the two events. But as they clearly recorded any barriers they faced, requests they were refused, and questions they never had answered in their meeting minutes, they were logging evidence of the bad faith behaviour and reneging on collaboration by the SHEG.

Beyond unsympathetic SHEG staff like Mitchell, WHO delegates, some of whom were feminists themselves, contributed to a culture of indifference surrounding the SWHF. Early meeting minutes from July 1982 recorded a conversation between the SWHF co-ordinating committee and “WHO representative, Ilona Kickbusch and her assistant Rena Freeman” where “it was accepted that the Conference and the Fair were interlinked.”^50^ But this cordial alignment was soon eclipsed by what SHWF organisers described as “early difficulties in achieving any kind of consensus” as the “‘proud-to-be-professional’ women” gradually left organising conversations between events.^51^ Margaretta Jolly’s work has shown how “the free marketization of Western economies in the 1980s propelled a new generation of professional women who held few feminist allegiances but seemed to demonstrate that women were now ‘liberated’” (Jolly, 2020, p. 307). She explains that while feminist women held positions of power, their ascension through the glass ceiling required them to buy into institutional beliefs and frameworks, which intensified debates about how to achieve change from within or outside of cultural systems and institutions. The WHO women working on the WAHC, like Ilona Kickbusch, had backgrounds in the “women’s and health movements.”^52^ But, in line with Jolly’s analysis, the WHO staff working on the WAHC felt the best way to effect change was from within their own institution. The WHO’s reluctance to compromise on their vision for the WAHC suggests the WHO staff positioned themselves as providing a service to women. But when the SWHF organisers’ vision no longer aligned with the WHO delegates’ own ideas of women’s health liberation, the proudly professional women of the WHO left the conversation. As Mindry’s concept of the “imagined feminine global family” (2001, p. 1189) suggests, the inability of the WHO delegates to see past their own institutional methods for change likely meant that the WHO feminists framed the SWHF organisers as obstructing their own empowerment. What started as a collaboration turned into a culture of indifference towards the SWHF, as the delegates from the WHO maintained professional borderlines and excluded grassroots participation in the planning and attendance of the conference.

The breakdown of the collaboration between the SWHF and the WAHC, and more specifically the SHEG, was bitter-sweet for Yvonne and the organisers of the fair. Yvonne remembered, “I felt I’d let these women down that I couldn’t make that [a Scottish women’s conference] happen.”^53^ But the SWHF would not have happened without her advocacy for Scottish women’s representation and her work to fund and resource the organising committee. Despite Yvonne’s best efforts, over the 18 months of planning for these events, the health fair organisers were increasingly left to their own devices. Yvonne’s story originally situates the origins of the SWHF as a dismissive compromise from the men at the SHEG. And oral histories and archival files reveal that SHEG and WHO staff failed to recognise the SWHF as a legitimate event with parallel status to the WAHC. But the growing distance between the SWFH and the WAHC also left the organisers of the fair unencumbered by institutional oversight, allowing them to promote their own vision for women’s health beyond the limits of health promotion by the SHEG and the WHO. Towards the end of her interview, as she reflected on the SWHF, Yvonne said,

I think at the end of the day, the fair was the right thing because it didn’t have the hand of WHO on it. It allowed all those people, like Penny [Richardson], like Jean [Malcolm], like Amanda [Amos], to actually do what the hell they wanted to do in that context. There probably wouldn’t have been that freedom if it had been a formal conference organised and satisfying the requirements of people at the top end.^54^

And the SWHF organisers did take the space to assert their autonomy from the WAHC. Frustrations mounting, by early May 1983, the SWHF press statement explicitly noted that the health fair was “linked with the SHEG/WHO Women and Health conference” and had been “undertaken with backing from SHEG” but had “otherwise been carried out independently by a co-planning group of women sharing a joint concern and interest in women’s health.”^55^ The increasing independence from the SHEG created space for the organisers of the fair to push back against what they saw as a top-down, medicalised, and universalised women’s health agenda, to host a health fair designed by and for Scottish women, and to assert their expertise in women’s health within their communities.

## Conclusion: The strategies and consequences of collaboration

The professional and feminist divides embedded within the history of the SWHF illuminate the motivations for and complexities of collaboration. Notions of expertise and knowledge clashed as Yvonne, other members of the SHEG, WHO professionals, and Scottish women’s health organisers all came to the proverbial table with their own agendas for women’s health in Scotland. SHEG employees were motivated to bolster the department’s status by collaborating on the WAHC and gaining WHO affiliate-centre status.^56^ The WHO representatives claimed to be drawn to the innovative health promotion happening at the SHEG and that they saw this partnership as an opportunity to enhance European health education agendas.^57^ And the SWHF organisers leveraged their link with the WAHC and SHEG funds to host an inclusive event that represented their own philosophies and approaches to bettering women’s health. Alongside these collective motivations, individuals strategised on the ground to mobilise their own ideas about women and public health in Scotland. As head of the SHEG, Stanley Mitchell’s objections to the *Well Woman* book, public argument with Yvonne at the WAHC, and hands-off approach to working with the SWHF organisers suggest his want for WHO affiliate-centre status outweighed any of his actual concerns for women’s health issues.

Yvonne’s advocacy for Scottish women to be included in the women’s health conference pushed the SHEG to sponsor a second, Scottish-women-run event in May 1983. She astutely navigated the professional and feminist boundaries as she worked on both events, lobbying for SHEG funds and resources for the SWHF and maintaining the, albeit thin, association between the WHAC and SWHF throughout 1982 and 1983. Yvonne was put in the position of an unsteady collaboration, and her story personifies the struggle for grassroots women’s health initiatives to be taken seriously within health institutions.

Yvonne’s work allowed others in the Scottish women’s health movement to execute their own vision for women’s health beyond the institutional and exclusive conference in Peebles. While the SWHF and the WAHC remained “associated” events, archival materials from the SWHF organising committee record increasingly fraught working relationships between themselves, the WAHC, and the SHEG in particular. By the end of the events, the SWHF organisers reflected in a report, “we were trying to balance in between officialdom and community groups but maybe [balancing] two different messages (i.e. one to each of the latter) is not an easy game to play!”^58^ SWHF organisers quickly realised that the collaboration with the SHEG and the WHO was in name only, that the WAHC organisers underestimated the SWHF as an opportunity for impact, and that Scottish women would be underrepresented. Thus, they shrewdly utilised SHEG sponsorship to bring thousands of Scottish women into the conversation about their own health and healthcare in May 1983. The SWHF organisers recording, and later preserving, their dissatisfaction with the SHEG’s gatekeeping of information and resources and the WHO’s dismissal of their contribution to the women’s health events of 1983 was also a political act. By explicitly outlining the fraught collaboration with the WAHC, the SWHF organisers claim the success of the fair as their own. In doing so, they rightfully claimed their place as major players, alongside national and global health institutions, in the 1983 women’s health events in Scotland.

## Notes

1 Yvonne assembled an organising committee of several women’s health organisers in Edinburgh, including Jo Burns, Fiona Macintyre, Jean Malcom, Linda Headland, Sarah Boyle, Fay Jacobsen, Jane Jones, Amanda Amos, Barbara Orton, Lyn Coutts, and Pauline Williams. SWHF meeting minutes, July–December 1982, GD31_8_1, Lothian Health Service Archives (LHSA), Edinburgh, UK. Yvonne Bostock, interview, 5 April 2024 (hereafter, YB, 2024).

2 YB, 2024.

3 SWHF Newsletter, November 1982; WHF Proposal, 22 June 1982, LHSA SWHF files.

4 SWHF meeting minutes, July–December 1983, GD31_8_1, LHSA; SHWF meeting minutes, January–June 1983, GD31_8_2, LHSA.

5 Draft report of SWHF, 6 July 1983, GD31_9_2, LHSA.

6 Shiela McNamara, article on the SWHF and the WHO, ca. 1983, news clipping from files courtesy of Yvonne Bostock; SWHF meeting minutes, July–December 1983, GD31_8_1, LHSA; SHWF meeting minutes, January–June 1983, GD31_8_2, LHSA; “Your Health, Your Fair: Scottish Women’s Health Fair” pamphlet, ca. 1982, GD31_9_2, LHSA.

7 Amanda Amos, interview, 12 March 2024 (hereafter, AA, 2024); Jane Jones, interview, 16 April 2024 (hereafter, JJ, 2024); Adrienne Sillar, interview, 26 April 2024 (hereafter, AS, 2024).

8 YB, 2024.

9 AA, 2024; YB, 2024; see also “Why Scots Women Die Young,” ca. 1982-3, newspaper clipping, personal collection of Adrienne Sillar, curtesy of Adrienne Sillar.

10 YB, 2024; AA, 2024.

11 JJ, 2024.

12 AS, 2024.

13 AA, 2024.

14 Shiela McNamara, article on the SWHF and WHO, ca. 1983, news clipping from files provided by Yvonne Bostock.

15 JJ, 2024.

16 AA, 2024.

17 YB, 2024.

18 YB, 2024; AA, 2024.

19 YB, 2024.

20 YB, 2024.

21 Conference Women & Health Provisional Programme, 1983, RAS_W6_87_3_JKT1, World Health Organization Archives (hereafter, WHOA), Geneva, Switzerland.

22 YB, 2024.

23 YB, 2024.

24 YB, 2024.

25 YB, 2024.

26 YB, 2024; AA, 2024.

27 Conference Women & Health Provisional Programme, 1983, RAS_W6_87_3_JKT1, WHOA.

28 YB, 2024.

29 Conference Women & Health list of participants, 1983; Conference Women & Health Provisional Programme, 1983, RAS_W6_87_3_JKT1, WHOA.

30 YB, 2024.

31 YB, 2024.

32 YB, 2024.

33 YB, 2024.

34 YB, 2024.

35 YB, 2024.

36 Draft Report of SWHF, 6 July 1983, GD31_9_2, LHSA.

37 Draft Report of SWHF, 6 July 1983, GD31_9_2, LHSA.

38 Draft Report of SWHF, 6 July 1983, GD31_9_2, LHSA.

39 SWHF organisers, “Women’s Health – A Discussion Paper Produced by the Organising Group of the Scottish Women’s Health Fair, 27–29 May 1983,” in the Edinburgh Women’s Liberation Newsletter Special Issue on Women and Health, Vol. 4, November 1982, Glasgow Women’s Library, Glasgow, Scotland; “The Scottish Women’s Health Fair: Proposed Publicity Statement,” GD31_9_1, LHSA; Draft Report of SWHF, 6 July 1983, GD31_9_2, LHSA.

40 SWHF organisers, “Women’s Health – A Discussion Paper produced by the Organising Group of the Scottish Women’s Health Fair, 27–29 May 1983,” in the Edinburgh Women’s Liberation Newsletter Special Issue on Women & Health, Vol. 4, November 1982, Glasgow Women’s Library, Glasgow, Scotland; “The Scottish Women’s Health Fair: Proposed Publicity Statement,” GD31_9_1, LHSA; Draft Report of SWHF, 6 July 1983, GD31_9_2, LHSA.

41 SWHF organisers, “Women’s Health – A Discussion Paper Produced by the Organising Group of the Scottish Women’s Health Fair, 27–29 May 1983,” in the Edinburgh Women’s Liberation Newsletter Special Issue on Women and Health, Vol. 4, November 1982, Glasgow Women’s Library, Glasgow, Scotland; “The Scottish Women’s Health Fair: Proposed Publicity Statement,” GD31_9_1, LHSA; Draft Report of SWHF, 6 July 1983, GD31_9_2, LHSA.

42 SWHF organisers, “Women’s Health – A Discussion Paper Produced by the Organising Group of the Scottish Women’s Health Fair, 27–29 May 1983,” in the Edinburgh Women’s Liberation Newsletter Special Issue on Women & Health, Vol. 4, November 1982, Glasgow Women’s Library, Glasgow, Scotland; “The Scottish Women’s Health Fair: Proposed Publicity Statement,” GD31_9_1, LHSA; Draft Report of SWHF, 6 July 1983, GD31_9_2, LHSA.

43 SWHF meeting minutes, 6 July 1982, GD31_8_1, LHSA.

44 SWHF meeting minutes, 25 January 1983, 15 February 1983, 10 May 1983, 16 June 1983, GD31_8_2, LHSA.

45 SWHF meeting minutes, 25 January 1983 and 15 February 1983, GD31_8_2, LHSA.

46 SWHF meeting minutes, 15 February 1983, GD31_8_2, LHSA.

47 SWHF meeting minutes, 10 May 1983, GD31_8_2, LHSA.

48 Draft Report of SWHF, 6 July 1983, GD31_9_2, LHSA.

49 SWHF meeting minutes, 6 July 1982, LHSA; write up of SWHF, 16 June 1983, LHSA.

50 SWHF meeting minutes, 6 July 1982, GD31_8_1, LHSA

51 Write up of SWHF, 16 June 1983, LHSA; Draft Report of SWHF, 6 July 1983, GD31_9_2, LHSA.

52 “Biographies” in Conference Women & Health Provisional Programme, 1983, RAS_W6_87_3_JKT1, WHOA.

53 YB, 2024.

54 YB, 2024.

55 SWHF information pack for press, 3 May 1983, GD31-9-1, Cervical Smear Campaign Collection, LHSA.

56 YB, 2024.

57 YB, 2024.

58 Draft Report of SWHF, 6 July 1983, GD31_9_2, LHSA.
